# An investigation into the effects of soil and fastener-freezing on ground vibrations induced by high-speed train in frozen regions

**DOI:** 10.1038/s41598-022-16675-5

**Published:** 2022-07-20

**Authors:** Yuhao Peng, Qionglin Li, Zongping Chen, Haodi Zhang, Xiaozhen Sheng

**Affiliations:** 1 State Key Laboratory of Traction Power, Southwest Jiaotong University, Chengdu, Sichuan China; 2 School of Civil Engineering, Southwest Jiaotong University, Chengdu, Sichuan China; 3 School of Urban Railway Transportation, Shanghai University of Engineering Science, Shanghai, China

**Keywords:** Civil engineering, Mechanical engineering

## Abstract

With the expansion of high-speed railway network in the world, it is inevitable for railways to pass through seasonal frozen regions. Since in a seasonal frozen region the ground can have significantly different mechanical properties between the freezing season and the warm season, train-induced ground vibration is also season-dependent but it has not received enough attention up to now. This paper gives an investigation into the effects of soil and fastener-freezing on ground vibrations induced by high-speed train in frozen regions. Based on the well-established relationships between soil mechanical properties and freezing temperature, a frozen ground is shown to be still represented by a layered ground and therefore, previously developed models for predicting ground vibration generated by a train running along a track resting on a layered ground can be readily applied. The effects of low temperature on the dynamical properties of fasteners are also considered. Results show that, due to the increased Young’s modulus at freezing condition, the vibration level of a frozen ground near the track is lower than that of the non-frozen counterpart. However, well away from the track, the vibration level of the frozen ground is much stronger than that of the non-frozen one, mainly due to the much-reduced loss factor of the frozen ground, which results in slower attenuation of vibration with propagating distance. Results also show that, the difference in ground vibration between a frozen ground and its non-frozen counterpart is mainly caused by freezing of the ground. The emphasis of this study lies in making clear the characteristics of train-induced ground vibration in frozen regions and the differences between frozen and non-frozen regions, providing some new fundamental insights about this practical problem, which have significant engineering guidance and application value.

## Introduction

**Figure 1:**
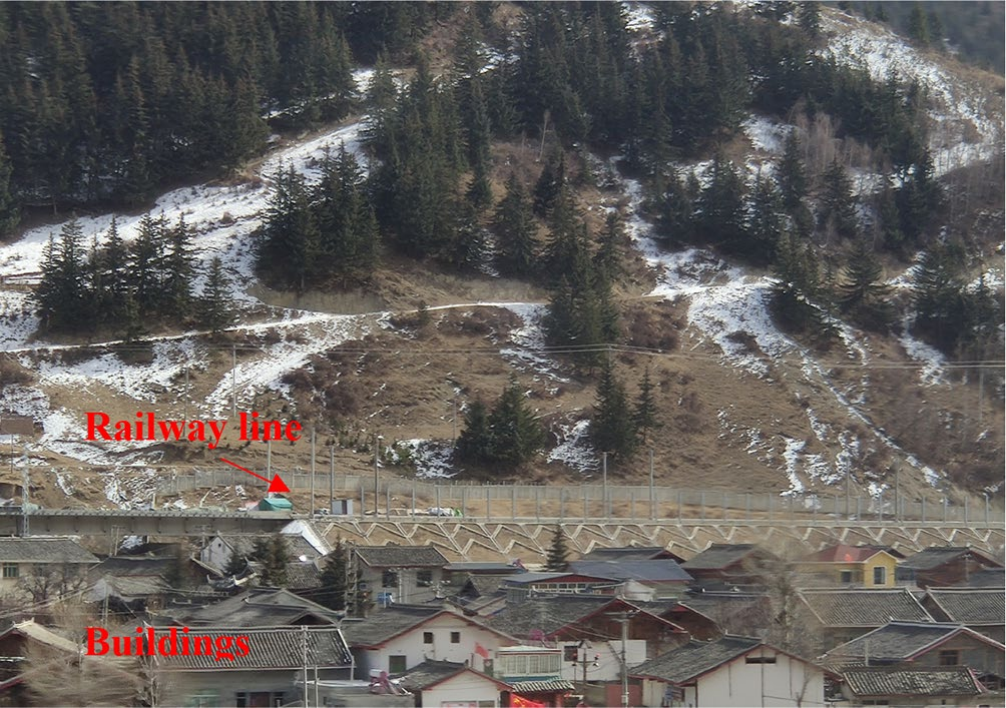
Railway line and surrounding buildings.

Railway is a transportation mode which not only has a large capacity, but also is environment-friendly compared with other transportation modes. Many countries are planning to build or develop their high-speed railway networks in various regions inevitably including some seasonal frozen ones. The global permafrost area accounts for about 20% to 25% of the land area, mainly distributed in the northern hemisphere. Trans-Siberians railway, Hudson Bay railroad and the planned Moscow-Kazan high speed railway in Russia all pass through frozen regions. Frozen regions are also widely distributed in China. A large area of permafrost is distributed in the Qinghai-Tibet Plateau and northern area of China, with an area of about 2.15 million km^2^, accounting for 22.4% of China’s land area^1^. There are several railway lines, such as the Qinghai-Tibet railway, Beijing-Harbin high-speed line, and Harbin-Dalian high-speed line etc., located in frozen regions and some inhabited areas or buildings are also near these railway lines, as shown Fig. 1. In these regions, the ground vibration caused by railway operations may vary significantly in different seasons. With the development of high-speed railways in frozen regions, ground vibration generated by railway operations in frozen regions becomes an important research topic.

When soil temperature drops below 0 °C, free water in the soil begins to freeze. Due to the effect of surface energy of soil particles, bound water adsorbed on the surfaces of the soil particles will also freeze as temperature reduces further. As a result, liquid water becomes ice crystals, and non-frozen soil becomes frozen soil. The ice crystals and the soil particles are cemented together, making the mechanical properties of the frozen soil significantly different from those of the non-frozen one. According to Refs.^2,3^, the mechanical properties of a frozen soil are very sensitive to temperature and soil/ice content. In some regions, the atmosphere temperature changes significantly with season, creating a cycle of soil-freezing in the winter and ice-thawing in the spring. Consequently, the mechanical properties of the soil and the dynamic characteristics of the ground vary significantly with season. It can be expected that, train-induced ground vibration is also season-dependant.

Vibrations of non-frozen grounds generated by railway operations or similar have been studied extensively. A large number of researchers have contributed to build analytical or numerical models to predict ground vibration. Metrikine^4^ and Vostroukhov^5^ studied the response of a beam periodically supported on a homogenous half-space and subject to a moving harmonic load. Otherwise, the ground is always assumed to be a horizontally layered structure. Haskell^6^ and Thomson^7^ proposed a transfer matrix method to analyse such a layered ground. Base on this method, Sheng et al.^8,9^ used the dynamic flexibility of a layered ground to predict ground vibration generated from a ballast track subject to a stationary or moving harmonic load. They simplified the ballast track to be uniform in the track direction and the coupling force (the normal stress on the track/ground interface) between the track and the ground is assumed to be uniformly distributed across the track width. A more realistic coupling allowing for a nonuniform normal stress is considered by Steenbergen and Metrikine^10^. Cheng^11^ and Auersch^12^ also simplified a slab track track/ground system to be invariant in the track direction to predict train-induced ground vibration. All the aforementioned models are, to some extent, analytic, requiring that the ground has a strict horizontally layered structure and each layer of the ground is homogeneous.

Yang and Hung^13,14^ used a numerical model called two-and-half dimensional (2.5D) finite/infinite element approach to predict train-induced ground vibration, and Wang et al.^15^ discussed several key problems in calculation of field response under moving loading using the 2.5D finite element method. Compared with the analytical models, the 2.5D approach has no strict restriction on the cross section of the track/ground system, but the structure and its material properties are still required to remain unchanged in the track direction, which is not always the case in practice. Due to the discrete support of rail and the discontinuity of track slabs, the track/ground system structurally is periodic in the track direction. For this periodic structure, based on Floquet transform, Clouteau, Degrande and Gupta et al.^16–18^ used a periodic finite element–boundary element approach to predict ground-borne vibrations generated by trains running in tunnels. Hussein et al.^19^ and Peng et al.^20^ also considered the periodicity of track/ground system and found that the bending modes of track slab can affect the frequency response of the ground. In Ref.^20^ the authors also investigated the condition under which the slabs can be replaced with an infinitely long Euler–Bernoulli beam.

When the track/ground structure is complex, for example, tunnels and buildings, three-dimensional finite element model is normally required. Yang et al.^21^ employed a two-step approach to predict vibrations induced by moving trains of a large-scale building co-constructed with a subway tunnel. In the first step, fastener forces are predicted using a time-domain vehicle-track interaction model. These fastener forces are Fourier-transformed into frequency components and the fastener force components at each frequency are, as the second step, applied to a three-dimensional finite element model comprising the track slabs, tunnel, ground and building. In order to better capture slab vibrations at medium–high frequencies, Yang et al.^22^ developed a dynamics model of the slab track on the basis of Reissner–Mindlin plate theory, to consider the effects of shear deformation and moment of inertia of the slabs. In addition to three-dimensional finite element models, Fiala, Lopes and Colaço et al.^23–25^ used sub-structured approaches to model the track-ground-building system, taking advantage of different techniques to deal with the different parts of the system, improving the computational efficiency. Numerical models for train-induced ground vibration have also been developed by Lombaert et al.^26^, Kouroussis et al.^27^, Xia et al.^28^ and Ju et al.^29^.

The above-mentioned researches are only for non-frozen regions. For train-induced ground vibration in frozen regions, not much work can be found in the literature published in English or Chinese. Several researchers have studies subgrade and ground vibration through experimental method. Ling et al.^30,31^ studied the subgrade vibration caused by trains in seasonally frozen regions of Daqing, China. Their results show that vibration attenuation of frozen ground differs from that of non-frozen one, although in both cases, subgrade vibration increases with train speed and load. Ling^32^ also studied track and ground vibration in permafrost regions along the Qinghai-Tibet railway through field monitoring. Jin^33^ measured the accelerations of piers and the ground surface generated by Changchun’s Light Rail Line 3 in winter and summer. The results show that the vibration of bridge pier in winter is lower than that in summer, but in the far field, the high frequency component of ground vibration is higher than that in summer because of the lower damping of the frozen ground. Xu^34^ used the 2.5D finite element method to predict frozen ground vibration induced by train for the purpose of studying near-field barrier isolation. Li^35^ proposed a thermal–dynamic coupled model which considers the plastic deformation of permafrost embankment to study the settlement of the embankment. Although through these studies some preliminarily understandings about the vibration characteristics and attenuation law of frozen ground have been developed, more comprehensive research from a theoretical point of view on this topic is still required. The various studies reviewed above on non-frozen grounds can provide a solid basis for this topic.

An investigation is presented in this paper into the effect of soil and fastener-freezing on ground vibrations induced by high-speed trains. It should be pointed out that, this paper focuses more on engineering significance and application value of train-induced ground vibration in frozen regions. Since the trains in Ledsgård exceed the critical speed of the ground, leading strong vibrations of the ground and trains and causing a series of social and economic problems^36^, the train-induced ground vibration has been concerned by many researchers. However, most researches about such an important topic are only limited to non-frozen regions and ignore the frozen regions, which are of equal importance. This paper based on the engineering perspective, aims to obtain some knowledge of the main characteristics of ground vibration in frozen regions in a clear and succinct way, providing some new fundamental insights about this problem. The work in this paper originates from a practical engineering problem and reminds researchers and railway engineers to consider not only the effects of soil-freezing, but also the seasonal dependence of ground vibration when evaluating the train-induced ground vibration in frozen regions, which have significant engineering guidance and application value.

In the investigation of this paper, the Chinese CRTS ΙΙΙ slab track and a typical high-speed moving train are considered. The CRTS ΙΙΙ slab track has been widely used in China and may also be used in many planned high-speed railway lines, including those in freezing regions. The ground is considered to have a horizontally layered structure and the dynamic flexibility method developed in Refs.^8,9^ is used to analyse the dynamics of the ground. A freezing atmosphere temperature makes the ground become a frozen ground and changes its material properties, resulting in a different layered structure. Relationships between frozen ground parameters (Young’s modulus, Poisson’s ratio, loss factor) and soil temperature reported in Refs.^35,37^ are employed in this paper and they are explained in detail in “Material and dynamic properties of a frozen ground and track”. For a frozen ground, soil temperature increases with depth, being the lowest (below 0 °C) on the ground surface and 0 °C at the so-called frozen depth. It is assumed that the material properties of soil further down are the same as the non-frozen one. To clearly delineate the temperature stratification of the ground caused by temperature gradient along the depth direction, the naturally stratified frozen ground is further divided into *N* horizontal layers with the same thickness. Determination of *N* for different surface temperatures and frozen depths is explained in “Material and dynamic properties of a frozen ground and track”. A freezing atmosphere temperature not only changes the ground parameters, but also has a significant effect on dynamic characteristics of fasteners. The effect of low temperature on the dynamic characteristics of the fastener is also discussed in "Material and dynamic properties of a frozen ground and track". Results are presented in “Results” section. The paper is concluded in “Conclusion”.

## Material and dynamic properties of a frozen ground and track

The material properties of a non-frozen ground change if it is frozen by low atmosphere temperature, especially in the vertical direction due to the temperature gradient formed in the soil. To analyse such changes, use is made of a non-frozen ground of which the parameters are listed in Table 1. The ground is formed by a layer of 3 m overlaying a homogeneous half-space. Three freezing atmosphere temperatures (− 5, − 10, − 20 °C) listed in Table 2 are considered to make the non-frozen ground (Ground 1 in Table 2) become a frozen ground. The corresponding frozen depths are also given in Table 2. Frozen depths are influenced by many factors and site-specific, and the values listed in Table 2 are typical according to Ref.^38^. Soil properties within the frozen depth depend on the negative temperature, but for the soil further down, the material properties are consistent with those of the non-frozen one.

**Table 1:** Parameters of the non-frozen ground.

Layer	P-wave speed (m/s)	S-wave speed (m/s)	Density (kg/m^3^)	Young’s modulus (MPa)	Poisson’s ratio	Loss factor	Depth (m)
1	250	120	1800	70	0.35	0.1	3
Half-space	732	299	2000	500	0.40	0.1	∞

**Table 2:** Surface temperatures and frozen depths of the grounds.

Grounds	1	2	3	4
Surface temperature / *T*_0_ (℃ )	≥ 0	− 5	− 10	− 20
Frozen depth / *h*_frozen_ (m)	0	0.5	1.5	2.5

In this section, the material properties of the frozen grounds are formulated first (“Material properties of the frozen ground as function of depth”). Use is made of formulas proposed in Refs.^35,37^. To make use of the layered ground theory presented in Refs.^8,9^, and at the same time to be able to describe the dependence of the material properties on depth, a frozen ground is divided into *N* horizontal and homogeneous layers with identical thickness. The layer number *N* is determined using dispersion curves and ground surface displacements due to circular and strip harmonic loads (“Stratifications for the frozen grounds”). Track parameters are discussed in “Dependence of the stiffness and damping of fastener system on temperature”.

### Material properties of the frozen ground as function of depth

When the temperature, *T*_0_, on the surface of a ground is below 0 °C, an upper part of the ground will be frozen. According to Refs.^39,40^, the temperature *T* in the soil may be assumed to increase linearly with depth *h* until it reaches 0 °C at the so-called frozen depth *h*_frozen_. Once the ground surface temperature and frozen depth are specified, the temperature gradient along the depth direction can be uniquely determined by

1 $$T = T_{0} (1 - h/h_{{{\text{frozen}}}} ),$$

where *T*_0_ < 0, *T* < 0, $0 \le h \le h_{{{\text{frozen}}}}$. The mechanical parameters of ground can be described as follows^35,37,41^

2 $$E = a_{1} + b_{1} \left| T \right|^{m} = a_{1} + b_{1} \left| {T_{0} } \right|^{m} (1 - h/h_{{{\text{frozen}}}} )^{m}$$

3 $$\upsilon = a_{2} + b_{2} \left| T \right| = a_{2} + b_{2} \left| {T_{0} } \right|(1 - h/h_{{{\text{frozen}}}} )$$

4 $$\eta = \eta_{\max } (a_{3} + b_{3} e^{{T/c_{3} }} ) = \eta_{\max } (a_{3} + b_{3} e^{{T_{0 } (1 - h/h_{{{\text{frozen. } } } } ) / c_{3} }} ),$$

where, *E*, $\upsilon$ and *η* are Young’s modulus (in MPa), Poisson’s ratio and loss factor of the ground respectively. *T* is the ground temperature (in °C) with *T* < 0. Constants *a*_*i*_, *b*_*i*_ (*i* = 1, 2), *c*_3_ and *m* are obtained by experiment. According to Refs.^35,37^, Eqs. (2)–(4) can be applied to clay soil and sandy soil. *a*_1_, *a*_2_ and $\eta_{\max }$ are Young’s modulus, Poisson’s ratio and loss factor of the non-frozen ground. For sandy soil used in this paper (the upper layer in Table 1), *b*_1_ = 41, *b*_2_ = − 0.005, *a*_3_ = 0.588, *b*_3_ = 0.412 and *c*_3_ = 4.233; for clay *b*_1_ = 27, *b*_2_ = − 0.008, *a*_3_ = 0.32, *b*_3_ = 0.69 and *c*_3_ = 2.18. For a given aqueous soil sample, these constants can be directly determined by experiments and the power *m* in Eq. (2) is usually taken to be 0.6^35,37^.

**Figure 2:**
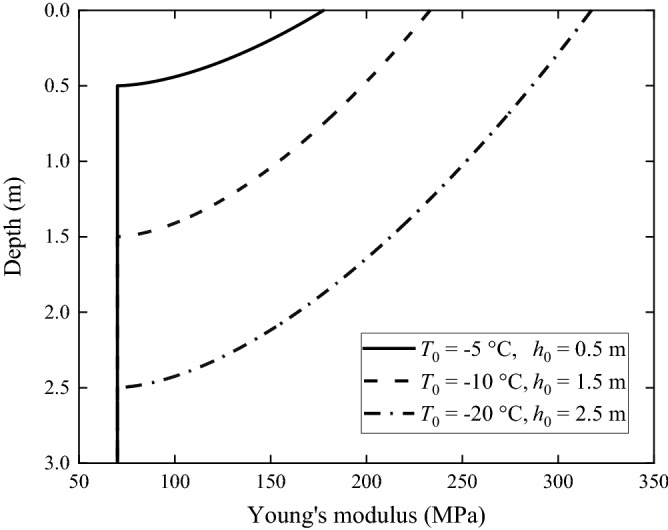
Young's modulus of the frozen ground as function of depth.

**Figure 3:**
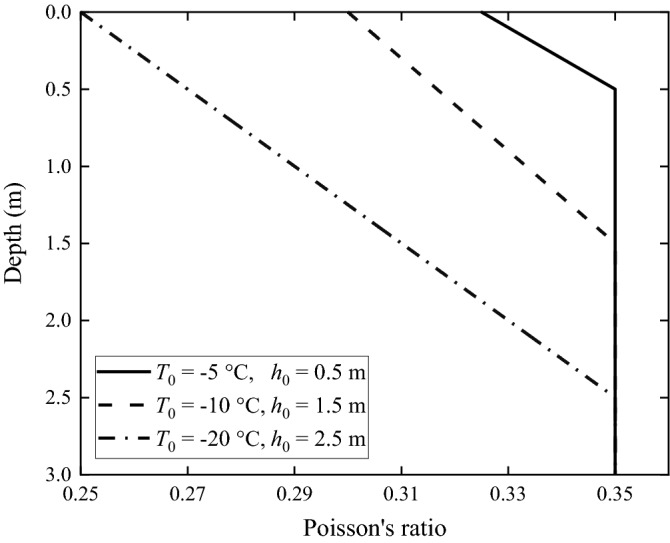
Poisson’s ratio of the frozen ground as function of depth.

**Figure 4:**
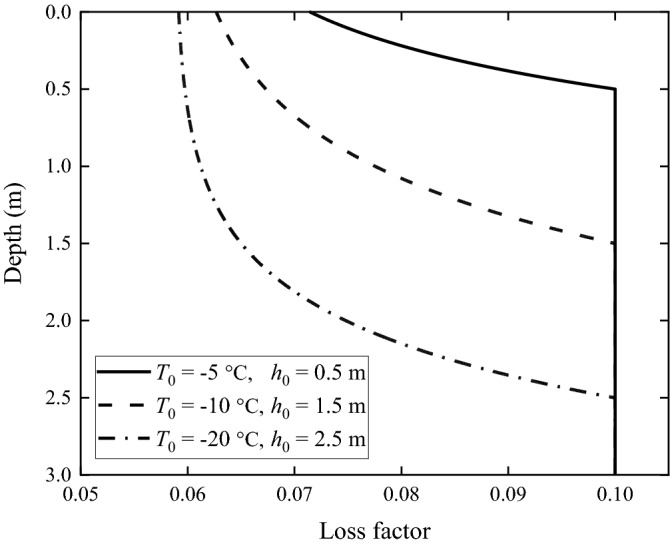
Loss factor of the frozen ground as function of depth.

According to Table 2, the frozen depths of the frozen grounds are less than 3 m, i.e., less than the thickness of the upper layer of the non-frozen ground. The relationships between material properties and depth of the upper layer are shown in Figs. 2, 3 and 4. It can be seen from Fig. 2 that, the Young's modulus of the frozen ground is higher than that of the non-frozen one, and the lower the local temperature is, the higher is the Young's modulus. When temperature is − 20 °C, the Young's modulus is 317.4 MPa, 4.5 times of that of the non-frozen ground (70 MPa). In Fig. 3, the Poisson's ratio of the frozen ground decreases as temperature reduces; when temperature is − 20 °C, the Poisson's ratio is 0.25, in contrast to 0.35 for the non-frozen ground. Figure 4 shows the loss factor of frozen ground which also decreases as temperature reduces. When the temperature is − 20 °C, the loss factor is only 0.059, approximately half that of the non-frozen ground.

**Figure 5:**
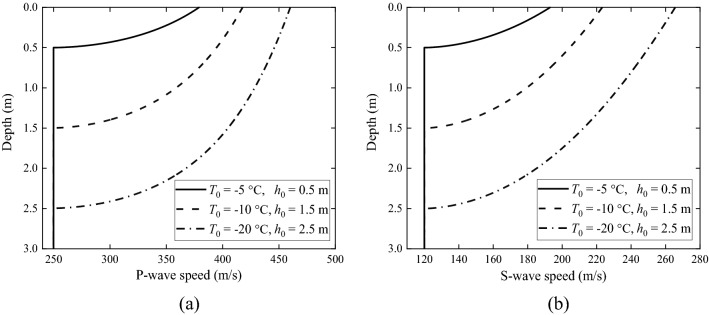
(**a**) P- and (**b**) S-wave speed of the frozen ground as function of depth.

Changes in Young's modulus and Poisson's ratio alter the P-wave and S-wave speeds of the ground, as shown in Fig. 5. In Fig. 5, P- and S- wave speeds increase significantly with the decrease of temperature, leading to different dynamic characteristics of the ground. For example, when temperature is − 20 °C, the S-wave speed increases to 265.5 m/s, more than twice of that of non-frozen ground. In summary, the material properties of a frozen soil are significantly affected by temperature; a lower temperature makes the soil stiffer, have smaller Poisson effect and less damped. Therefore, a frozen ground may exhibit significantly different dynamic characteristics from the corresponding non-frozen ground.

According to the Young’s modulus and Poisson's ratio given in Eqs. (2) and (3), the shear wave speed, *v*_*s*_, of ground with different frozen temperatures can be determined $\left( {v_{s} = \sqrt {\frac{E}{2\rho (1 + \upsilon )}} } \right)$. In order to verify the variation trends of soil material properties with temperature in Eqs. (2) and (3), the GDS bending element test system is used to measure the shear wave speed and shear modulus of frozen ground with different frozen temperatures, and the measured results are compared with those calculated by Eqs. (2) and (3).

**Figure 6:**
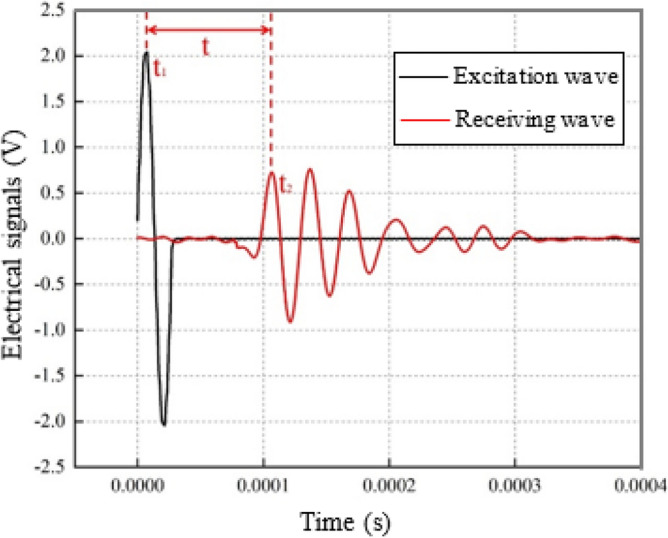
Determination of shear wave propagation time.

The operating principle of the GDS bending element test system can be laconically introduced through Fig. 6. The soil sample has two ends. During the test, the shear wave (sine wave) is emitted from the excitation terminal (one end of the soil sample) and the vibration signal is measured at the receiving terminal (the other end of the soil sample). Therefore, the propagation time *t* of the shear wave in the soil sample is obtained according to corresponding times, *t*_1_ and *t*_2_, of the first wave crests of signals measured at excitation terminal and receiving terminal, as indicated in Fig. 6. The shear wave speed, *v*_*s*_, is finally determined by the propagation distance, *d*, and the propagation time, *t*, of the shear wave, i.e., *v*_*s*_ = *d*/*t*, where *t* = *t*_2_ − *t*_1_.

**Figure 7:**
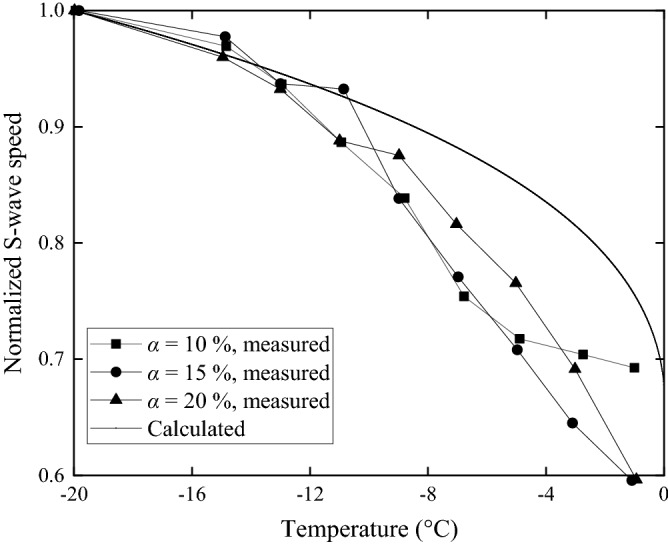
Relationships between frozen temperature and shear wave speed of ground with different soil water content (soil dry density *ρ*_d_ = 1700 kg/m^3^).

**Figure 8:**
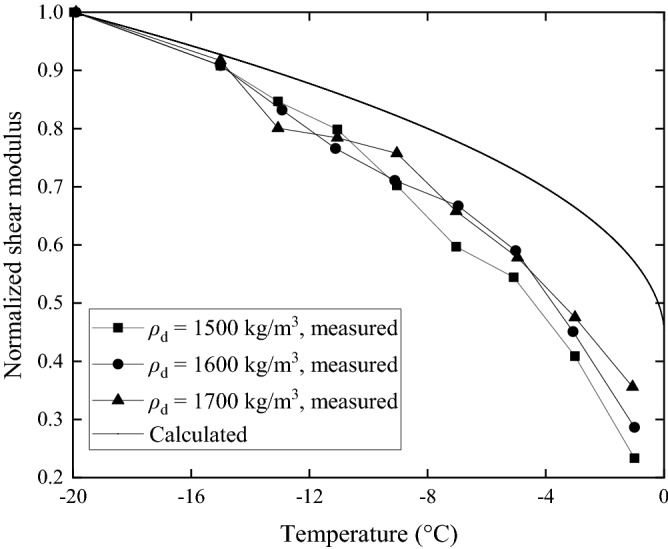
Relationships between frozen temperature and shear modulus of ground with different soil dry density (soil water content *α* = 20%).

Before the test, the soil samples are quickly frozen at − 30 °C, and then kept at the specified frozen temperature for 12 h. Once the shear wave speed, *v*_*s*_, is obtained through the test, the shear modulus, *G*, can be therefore determined by formula *G* = *ρ*_·_*v*_*s*_^2^, where *ρ* is the density of soil sample. It should be pointed out that the ground parameters are affected by many factors, such as water content, dry density and temperature of the soil. Therefore, this paper only compares the variation trends with temperature of soil shear wave speed and shear modulus measured by tests and calculated by Eqs. (2) and (3). The shear wave speed versus temperature is shown in Fig. 7, when dry density of soil is 1700 kg/m^3^ and the soil water contents, *α*, are 10%, 15% and 20%, respectively. While Fig. 8 shows the relationship between soil shear modulus and temperature. The dry densities of soil, *ρ*_d_, are 1500 kg/m^3^, 1600 kg/m^3^ and 1700 kg/m^3^, with the corresponding soil water content equal to 20%. The minimum temperature considered in the figures is − 20 °C, so the shear wave speed and shear modulus are normalized by those at − 20 °C. With the decrease of negative soil temperature, the shear wave speed and shear modulus of soil increased significantly. In Figs. 7 and 8, the variation trends obtained from the tests are basically consistent with those calculated by Eqs. (2) and (3). It can be considered that the relationships between soil materials and temperature in Eqs. (2) and (3) are appropriate.

### Stratifications for the frozen grounds

**Figure 9:**
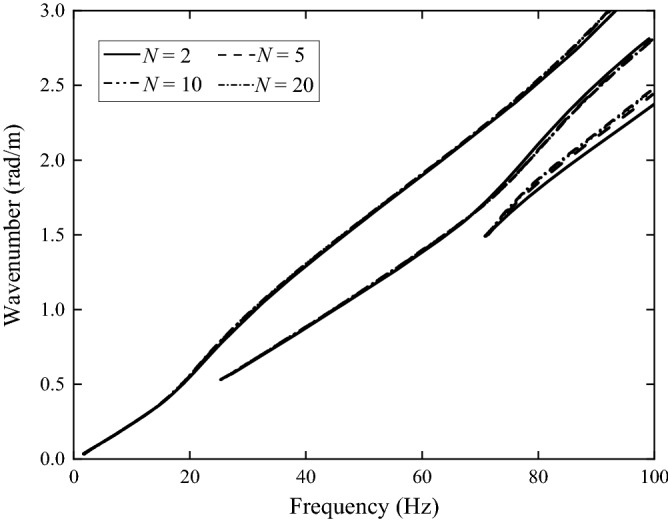
Dispersion diagram of frozen ground with different layers (*T*_0_ = − 20 °C, *h*_frozen_ = 2.5 m).

**Figure 10:**
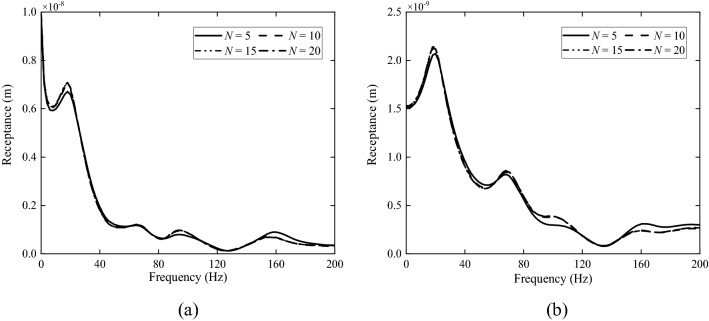
The vertical displacement of the ground surface at the centre of the loading area predicted for the third frozen ground with different layers. (**a**) Due to a vertical strip load; (**b**) due to a vertical circular load.

In this paper, the layered ground theory presented in Refs.^8,9^ is used to analyse the frozen grounds. However, this approach requires that the ground has a horizontally layered structure and each layer is homogeneous. Due to freezing, the soil in the upper layer, as shown in Figs. 2, 3 and 4, becomes non-homogeneous, and therefore, for the layered ground theory to be applicable, the frozen upper layer is further divided into *N* sub-layers having the same thickness and the thickness is sufficiently small (or *N* is sufficiently large) that each sub-layer can be approximated to be homogeneous (of course, the frozen upper layer may be divided into sub-layers of different thicknesses. Here a single thickness is used for making things easy. From Figs. 2, 3 and 4 it can be seen that, when all the sub-layers are of the same thickness, variation in material property within a sub-layer is smaller if it is located closer to the ground surface. This makes the response of the ground surface to excitations also on the ground surface less sensitive to the non-homogeneity of lower sub-layers). However, a too large *N* leads to significant reduction in calculation efficiency. Therefore, it is important to choose an appropriate value for *N* to adequately reflect the variations of soil properties with depth and in the meantime limit the calculation time as short as possible. *N* may be determined using dispersion curves and ground responses to a harmonic load. Take Ground 4 in Table 2 as example (the surface temperature *T*_0_ = − 20 °C and the frozen depth *h*_frozen_ = 2.5 m). Figure 9 shows dispersion curves of the frozen ground with different layers. The dispersion curves of the frozen ground are obtained in the frequency-wavenumber domain and the details of the method for the determination of dispersion curves are summarized in detail in Chapter 3 of Ref.^46^. It can be seen from Fig. 9 that the dispersion curves are not particularly sensitive to the layer number *N*: in terms of dispersion curve, *N* = 5 is sufficient. When *N* = 5, the maximum relative variation of the layers in Young’s modulus is 331.8% and that in Poisson’s ratio and loss factor is 25.7% and 40.6% respectively. Figure 10 shows the vertical displacement of the ground surface at the centre of the loading area for different layer number *N* under vertical strip load (width = 3.1 m) and circular load (diameter = 3.1 m)^8^, where 3.1 m is exactly the contact width between the track and ground. The result converges as *N* approaches 5. Therefore, in what follows, the number of sub-layers *N* is taken to be 5. It has been checked that this value is also applicable to all other frozen grounds defined in Table 2.

**Figure 11:**
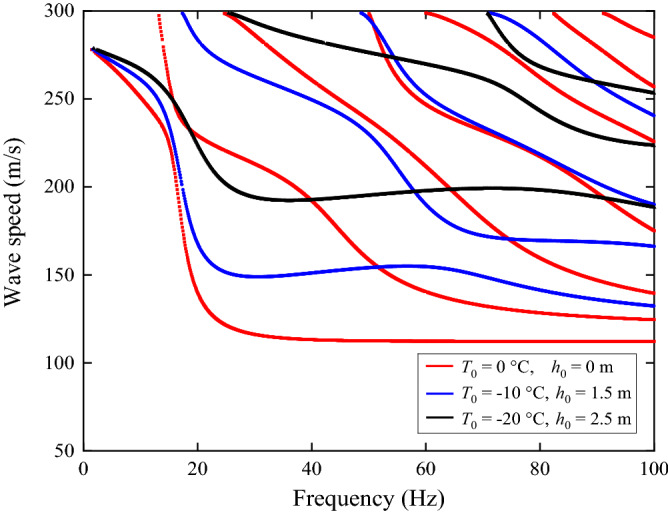
Dispersion curves of the frozen and non-frozen ground.

Figure 11 shows the dispersion curves of the frozen grounds defined in Table 2. From Fig. 11 it can be seen that, the number of propagation waves within 100 Hz become fewer as freezing temperature reduces. This is because, as freezing temperature reduces, a propagating wave cuts-on at a higher frequency. For the condition of surface temperature *T*_0_ = − 20 °C and frozen depth *h*_0_ = 2.5 m, the first three cut-on frequencies are 0 Hz, 25.3 Hz and 70.6 Hz while those of the non-frozen ground are 0 Hz, 13.1 Hz and 24.7 Hz. The dispersion curve of the frozen ground exhibits the higher propagation wave speed than non-frozen ground, also leading a higher critical speed. The critical speed will be further analysed in “Critical speed” section.

**Figure 12:**
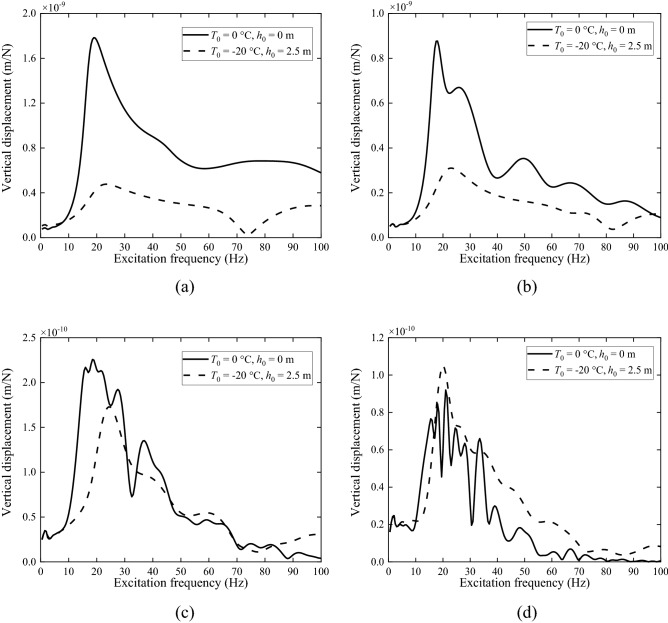
Vertical displacement of ground at (**a**) *y* = 5 m, (**b**) *y* = 10 m, (**c**) *y* = 20 m and (**d**) *y* = 30 m along the *y*-axis due to a unit point load acting at *x* = 0 and *y* = 0 with different excitation frequencies.

In addition, ground displacements are also calculated for the frozen ground and non-frozen ground with *T*_0_ = − 20 °C, *h*_0_ = 2.5 m induced by a unit point load with different excitation frequencies acting at *x* = 0 m and *y* = 0 m (transfer functions). The observation points are along the *y*-axis and located at *y* = 5 m, 10 m, 20 m and 30 m, as shown in Fig. 12. The resonance frequencies of the ground can be found in Fig. 12a at just below 20 Hz for the non-frozen ground and above 20 Hz for the frozen one. The resonance is much sharper for the non-frozen ground. Because the non-frozen ground has lower modulus and larger damping, which gives discrepant attenuation of the ground response at different frequencies, the ground response curve is more complicated with the increase of propagating distance, exhibiting more peaks. Figure 12 clearly depicts the process in which the response of frozen ground exceeds that of non-frozen ground with the increase of distance. In Fig. 12a,b, the responses of frozen ground are significantly lower than those of non-frozen one because of high modulus of frozen ground. In Fig. 12c, the response of frozen ground comes close to that of non-frozen ground and finally exceeds the response of non-frozen ground at most frequencies, as shown in Fig. 12d, because the frozen ground is less damped.

### Dependence of the stiffness and damping of fastener system on temperature

The stiffness and damping of fasteners are significantly affected by temperature. Especially when the temperature is close to the glass transition temperature of the rail pad material (polyurethane), they change dramatically, so it is necessary to consider the temperature-dependent properties of fastener systems when predicting train-induced ground vibration in frozen regions. However, for the frequency concerned in ground vibration (1–80 Hz for feelable vibration of human body and 20–250 Hz for ground-borne noise^42^), according to Refs.^43,44^, the stiffness can be approximately regarded as frequency independent.

**Figure 13:**
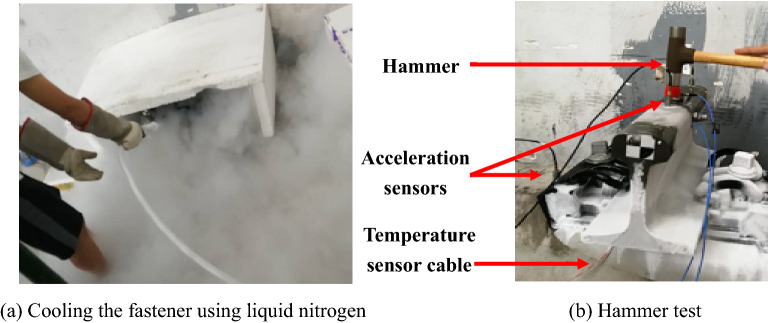
Measurement of fastener stiffness and damping at freezing temperatures. (**a**) Cooling the fastener using liquid nitrogen, (**b**) Hammer test.

Experiments are performed to obtain the temperature-dependent properties of the fastener system (Model WJ-8) and carried on a short rail fastened to a sleeper (Fig. 13). The rail is only 40 cm long so that it can be regarded as a rigid body in the concerned frequency range. The rail-fastener-sleeper system is enclosed with a container. Liquid nitrogen is poured into (Fig. 13a) the container to cool the fastener to required temperatures. Hammer testing (Fig. 13b) is then performed at specified temperatures (− 5 °C, − 10 °C, − 20 °C). The experimental modal analysis method is used to identify the dynamic stiffness and damping of fastener system.

Before the hammer test, a high sensitivity temperature transducer is installed near the fastener to measure the negative temperature at each time. The whole fastener system is put in a foam box and cooled with liquid nitrogen to − 50 °C for an enough long time, as shown in Fig. 13. Then stop using liquid nitrogen and use the harmer to excite the rail at the moment the temperature displayed by the temperature sensor reaching the required temperature. The force sensor on the hammer is used to obtain the load amplitude applied on the rail. The whole test process is enough short to ensure that the results have sufficient accuracy.

**Figure 14:**
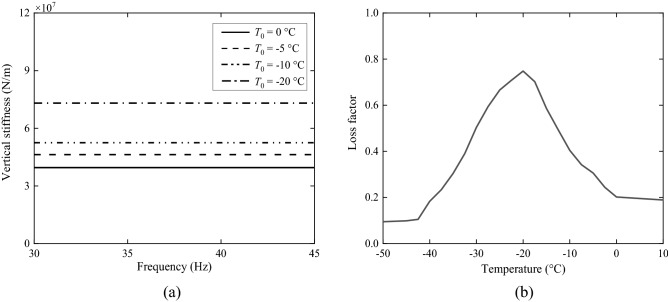
Experiment results of (**a**) vertical stiffness and (**b**) damping of WJ-8 fastener at different temperatures.

The stiffness of the fastener at different temperatures are plotted in Fig. 14a for the frequency range from 30 to 45 Hz. They are obtained by smoothing the measured results. Figure 14b gives the loss factor of the fastener at different temperatures. With the decrease of temperature, the loss factor first increases and then starts to decrease at the glass transition temperature. For the WJ-8 fasteners of CRTS III slab track, the glass transition temperature is around − 20 °C, a common temperature in the winter of many frozen regions. The measured vertical stiffness and loss factor of the fastener at − 5 °C, − 10 °C, − 20 °C and more than 0 °C are listed in Table 3. The maximum relative variation of fastener stiffness is 46.9% and that of fastener loss factor reaches 274%. In the track model, two rails are represented as a single beam with rail cross-sectional geometric quantities doubled. Consequently, the vertical stiffness of the fastener in Table 3 is multiplied by a factor 2. Besides, the consideration of viscous damping is frequently used in the literature and the loss factor always needs to be converted to viscous damping coefficient^45^. For a spring-mass system of single freedom with viscous damping, the frequency response function is

5 $$H(\omega ) = \frac{1}{{ - \omega^{2} m + {\text{i}}\omega c + k}},$$

**Table 3:** Dependence of WJ-8 fastener stiffness and damping on temperature.

Temperature (℃ )	≥ 0	− 5	− 10	− 20
Vertical stiffness of fastener (N/m)	3.95 × 10^7^ × 2	4.63 × 10^7^ × 2	5.25 × 10^7^ × 2	7.31 × 10^7^ × 2
Loss factor of fastener	0.2	0.306	0.404	0.748

where $\mathrm{i}=\sqrt{-1}$. *m*, *k*, *c* are mass, stiffness and viscous damping of the spring-mass-damper system, and *ω* is response frequency (in rad/s). Equation (5) can be further written as

6 $$H(\omega ) = \frac{1}{k}\left[ {\frac{{1 - \overline{\omega }^{2} }}{{(1 - \overline{\omega }^{2} )^{2} + (2\xi \overline{\omega })^{2} }} + {\text{i}}\frac{{ - 2\xi \overline{\omega }}}{{(1 - \overline{\omega }^{2} )^{2} + (2\xi \overline{\omega })^{2} }}} \right],$$

where $\xi = c/(2m\omega_{0} )$ and $\overline{\omega } = \omega /\omega_{0}$. $\omega_{0} { = }\sqrt {k/m}$ is the natural frequency of the spring-mass system. When the loss factor *η* (structural damping) are used, the stiffness of spring-mass-damper system becomes complex k^*^ = *k*(1 + i*η*). Equations (5) and (6) become

7 $$H(\omega ) = \frac{1}{{ - \omega^{2} m + k^{*} }} = \frac{1}{{ - \omega^{2} m + (1 + i\eta )k}} = \frac{1}{k}\left[ {\frac{{1 - \overline{\omega }^{2} }}{{(1 - \overline{\omega }^{2} )^{2} + \eta^{2} }} + {\text{i}}\frac{ - \eta }{{(1 - \overline{\omega }^{2} )^{2} + \eta^{2} }}} \right].$$

Comparing Eqs. (6) and (7), the loss factor can be converted to viscous damping by letting $2\xi \overline{\omega } = \eta$. For the track system, *k* and *m* are rail pad stiffness and mass of rail per unit length along the track. At resonance frequency *ω*_0_, the whole rail bounces on the rail pad^20^.

It should be pointed out that, wheel-rail forces are mainly affected by the track and the unsprung masses of the train, and therefore in this paper, suspension parameters of the train are assumed to be unchanged by temperature.

## Results

At freezing atmosphere temperature, not only a layer of soil near the ground surface will be frozen, the rail fastener system will also become stiffer. A stiffer rail fastener system may increase wheel/rail interactions, and in turn may change forces exciting ground vibration. A frozen ground is stiffer than its non-frozen counterpart, but according to Eq. (4) and Fig. 4, it is less damped. Therefore, it is not that straightforward to tell in which season (the frozen season or the warm season) the train-induced ground vibration is greater. Predictions of train-induced ground vibration based on seasonal parameters are necessary. This section presents some illustrating results from such predictions. Since the dependences of soil properties on temperature have already been dealt with in “Material properties of the frozen ground as function of depth”, this section starts with a description of the train and track (“Track and train parameters”). In “Critical speed”, the critical speed of track/frozen ground system is analysed. In “Train-induced ground vibration”, the train-induced ground vibration is predicted and the effects of soil-freezing and fastener-freezing are differentiated also in this section. During the investigation, we have strictly controlled the variables, i.e., the vehicle model, track model and irregularity spectrum are consistent when predicting the ground response under different freezing temperatures (wheel-rail forces are mainly affected by the track and the unsprung masses of the train, so the vehicle model are assumed to be unchanged by temperature) and only the properties of soil and fastener change with different freezing temperatures.

### Track and train parameters

**Figure 15:**
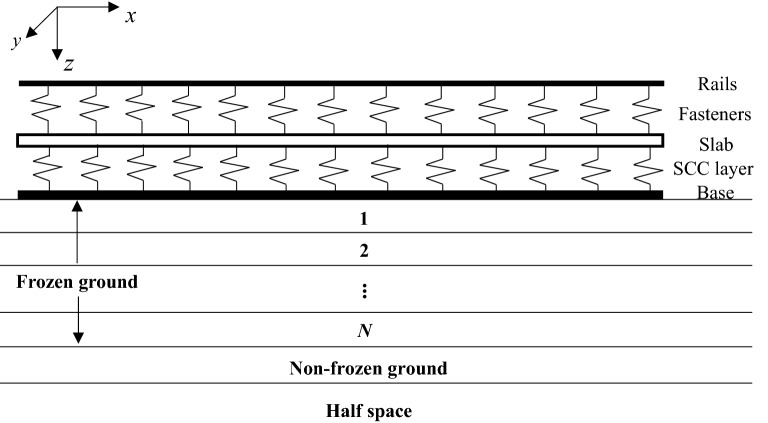
A sketch of the CRTS III slab track/frozen ground and the coordinate system.

The track and the mathematically layered frozen ground are depicted in Fig. 15 and the track parameters are listed in Table 4. The track is a multilayer structure (two rails, fastener systems, track slabs, a self-compacting concrete (SCC) layer and a concrete base). Based on the research of Ref.^20^, the track slabs can be replaced with an infinitely long beam in the frequency range concerned in ground vibration (1–80 Hz for feelable vibration of human body and 20–250 Hz for ground-borne noise^42^) due to the high stiffness of the SCC layer and the relatively low (even at as low as − 20 °C) stiffness of the fastener system. Therefore, in this paper, two rails, track slabs and base are represented as, respectively, three infinitely long Euler–Bernoulli beams which are connected by fastener systems and SCC layer represented as continuously distributed springs. The track/ground system is simplified to be invariant in the track direction. The ground is analysed based on the transfer matrix method proposed by Haskell and Thomson^6,7^ and the track and ground system are coupled in the frequency-wavenumber domain through the displacement continuity condition. The mathematical formulations have been thoroughly derived in Refs.^8,9^ and summarized in detail in Chapter 2 of Ref.^46^. Regarding the track/ground interaction, as done in Ref.^10^, the interface between the track and ground is divided into twelve identical strips (only normal stress in the interface is considered and in each strip the stress is assumed to be uniform in the *y*-direction) to make the track/ground coupling closer to reality.

**Table 4:** The parameters of the CRTS III slab track at normal temperature.

Mass of rail beam per unit length	*m*_R_ = 60.72 × 2 kg/m
Bending stiffness of rail beam	*EI*_R_ = 6.62 × 10^6^ × 2 N**·**m^2^
Loss factor of rail	η_R_ = 0.01
Vertical stiffness of fastener	*k*_P_ = 3.95 × 10^7^ × 2 N/m
Loss factor of rail pad	η_P_ = 0.2
Sleeper spacing	*l*_S_ = 0.58 m
Mass of track slab per unit length	*m*_S_ = 1250 kg/m
Bending stiffness of track slab	*EI*_S_ = 6 × 10^7^ N**·**m^2^
Loss factor of track slab	η_S_ = 0.1
Vertical stiffness of SCC layer per unit length	*k*_C_ = 7.78 × 10^9^ N/m^2^
Loss factor of SCC layer	η_C_ = 0.2
Mass of bed plate per unit length	*m*_B_ = 2325 kg/m
Bending stiffness of bed plate	*EI*_B_ = 2.27 × 10^8^ N**·**m^2^
Loss factor of base	η_B_ = 0.1
Width of track/ground interface	η*b* = 3.1 m

A typical Chinese high-speed train consisting of 8 vehicles is considered. Vehicle parameters are determined by referring to Ref.^47^ and listed in Table 5. All the vehicles are approximated to be identical to each other. Each vehicle is modelled as a multi-rigid body system vibrating in the vertical plane containing the track central line. Vehicles are coupled by the track only.

**Table 5:** Vehicle parameters.

Mass of the vehicle body	*m*_C_ = 38,936 kg
Pitching moment of inertia of the vehicle	*J*_C_ = 1.7 × 10^6^ kg·m^2^
Length of the vehicle body	*l*_V_ = 24.5 m
Mass of the bogie	*m*_B_ = 2328 kg
Pitching moment of inertia of the bogie	*J*_B_ = 2602 kg·m^2^
Distance between bogie centres	*l*_B_ = 17.375 m
Mass of the wheelset	*m*_W_ = 1639 kg
Contact stiffness	*k*_H_ = 0.5 × 10^9^ N/m
Distance between axles	*l*_A_ = 2.5 m
Vertical stiffness of the primary suspension	*k*_P_ = 0.75 × 10^6^ N/m
Vertical viscous damping of the primary suspension	*c*_P_ = 10 kN·s/m
Vertical stiffness of the secondary suspension	*k*_S_ = 0.2 × 10^6^ N/m
Vertical viscous damping of the secondary suspension	*c*_S_ = 10 kN·s/m

**Figure 16:**
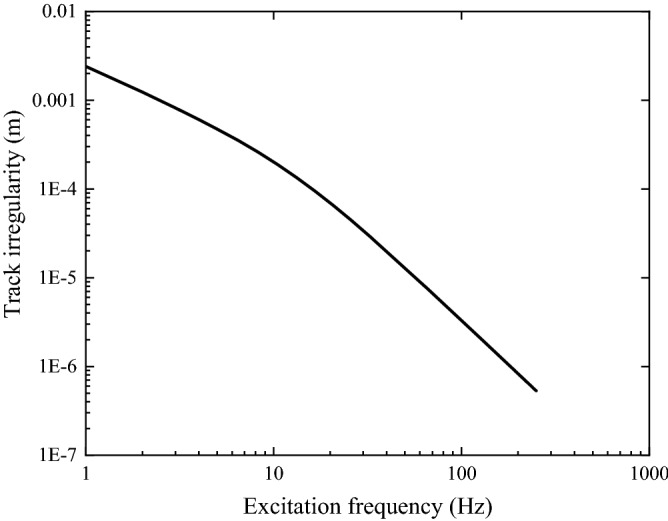
Track irregularity in the frequency domain (train speed: 100 m/s).

The spectrum of the track irregularity used in this paper is shown in Fig. 16. It is plotted against frequency (train speed is 100 m/s) and derived from the PSD (power spectral density) of track irregularity following an FFT-based method described in Chapter 3.5 of Ref.^48^. The irregularity spectrum is the German spectrum for train speeds beyond 250 km/h^48^.

### Critical speed

**Figure 17:**
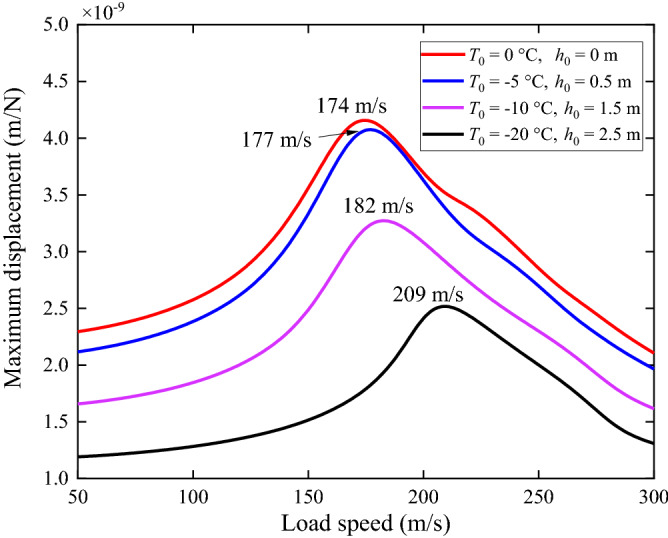
Load speed-maximum displacement curves of track/frozen ground system.

For the track/ground system with different surface temperatures and frozen depths, Fig. 17 shows the load speed-maximum displacement curves along the track center line. The critical speeds (peak speeds) in the figure are 174 m/s (red line), 177 m/s (blue line), 182 m/s (purple line) and 209 m/s (black line), respectively. As summarized from Fig. 2, with the decrease of the ground surface temperature, the ground becomes stiffer, then for a given frequency, the corresponding wavenumber also decreases and the wavelength of the propagating wave becomes longer which results in a higher phase velocity ($c_{{{\text{phase}}}} = 2{\uppi }f_{0} /\beta_{0}$) and critical speed. The critical speed of the track/ground system with *T*_0_ = − 20 °C and *h*_0_ = 2.5 m is 35 m/s higher than that of non-frozen one and it is beneficial to ground vibration.

### Train-induced ground vibration

The vehicle in this paper is modelled as a multi-rigid body system. The train and track/ground system are coupled based on the receptance matrices of the train and rail at the rail/wheel contact points and the wheel-rail interaction forces are solved in the frequency domain. The train-induced ground displacement spectrum is calculated to be the product of that due to a unit harmonic force moving along the rail and the wheel-rail interaction force^9^. More details can be found in Refs.^46^.

#### One-third octave band acceleration level spectrum

(1) Both the effect of freezing temperature on soil and that on fastener are considered.

**Figure 18:**
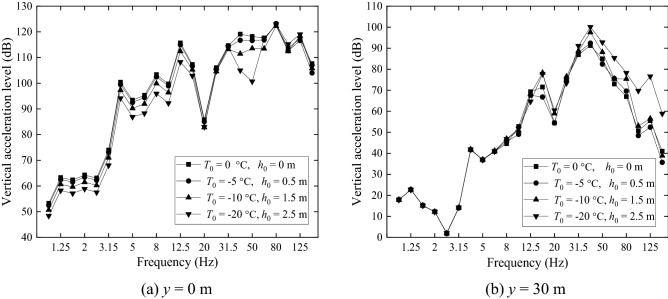
Ground vibration acceleration level in one-third octave bands at *x* = 0, (**a**) *y* = 0 m and (**b**) *y* = 30 m.

One-third octave band acceleration levels in dB of the four grounds are shown in Fig. 18 for two locations on the ground surface, one being at *x* = 0 and *y* = 0 (Fig. 18a) and the other at *x* = 0 and *y* = 30 m (Fig. 18b). At *x* = 0 and *y* = 0, the non-frozen ground has a higher acceleration level while the acceleration level of the frozen ground with *T*_0_ = − 20 °C *h*_frozen_ = 2.5 m is lower than other three grounds at most frequencies. From Fig. 18b it can be seen that, for central frequencies lower than 20 Hz, the difference in ground vibration caused by freezing temperature is small. However, for higher frequencies, it is seen that a lower temperature causes a higher vibration level. The maximum difference reaches 21.1 dB at 125 Hz, although this frequency is relatively high for the frequency concerned in ground vibration.

(2) Only the effect of freezing temperature on soil is considered.

**Figure 19:**
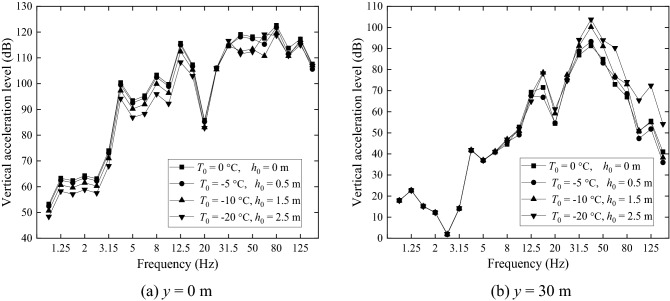
Ground vibration acceleration level in one-third octave bands at *x* = 0, (**a**) *y* = 0 m and (**b**) *y* = 30 m. Only the changes of ground parameters caused by freezing temperature is considered.

Figure 19 shows the acceleration levels of the four grounds at *x* = 0, *y* = 0 (Fig. 19a) and *x* = 0, *y* = 30 m (Fig. 19b), predicted with soil-freezing considered only (i.e., the stiffness (3.95 × 10^7^ × 2 N/m) and loss factor (0.01) of the fasteners at warm temperature are used). Figure 19a shows that, the non-frozen ground has a larger acceleration level than the frozen ones at most frequencies. This is mainly due to the increased Young’s modulus of the frozen grounds. However, in the far field (Fig. 19b), vibrations of the frozen grounds exceed those of the non-frozen one for frequencies above 12.5 Hz, although the frozen grounds are stiffer than the non-frozen one. The maximum difference reaches 16.85 dB at 125 Hz. This may be explained by the facts that the frozen grounds are less damped than the non-frozen one and vibration decay with distance of the frozen ground is smaller than the non-frozen one, as illustrated below.

**Figure 20:**
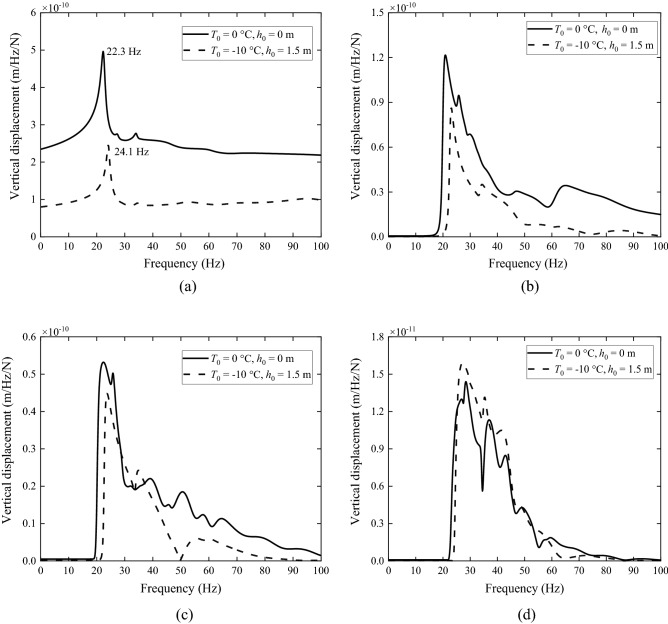
Spectra of the ground surface at (**a**) *y* = 0 m, (**b**) *y* = 5 m, (**c**) *y* = 15 m and (**d**) *y* = 20 m along *y*-axis due to a 40 Hz load moving along the *x*-axis at 100 m/s.

To give an example, Fig. 20 shows the vertical displacement spectra of the ground surface due to a 40 Hz unit harmonic load moving at 100 m/s along the *x*-axis (the track direction) for surface temperature equal to 0 °C and − 10 °C given in Table 2. Observations are made along the *y*-axis (the direction perpendicular to the track) on the ground surface from *y* = 0 m to 20 m. As shown in Fig. 20a, since the observation point is on the *x*-axis where the load is applied, the amplitude of the displacement spectrum decreases monotonically with reducing atmosphere temperature for almost every frequency considered. This is caused by the fact that the Young’s modulus of the frozen layer increases as temperature decreases. However, when an observation location is away from the *x*-axis (at *y* = 20 m), the response may be affected by wave propagations. As temperature decreases, the loss factor also decreases, making vibration decay become smaller with propagating distance. As a result, the effect of freezing atmosphere temperature becomes much more complicated for observation points away from the *x*-axis: the response of a frozen ground can be similar to that of the non-frozen one for some frequencies while greater for other frequencies. The lower Doppler frequency, *f*_L_, can be determined by that

8 $$f_{L} = \frac{{f_{e} }}{1 + c/v},$$

where *f*_e_ is excitation frequency and *c* is load speed. *v* denotes the phase velocity of characteristic wave of the ground, which is frequency dependent^49^. As indicated in Fig. 20a, the lower Doppler frequency is 22.4 Hz for the non-frozen and 24.1 Hz for the non-frozen soil.

If the vertical vibrational displacement of the ground surface at time *t* and position (*x*, *y*) is denoted by $w(x,y,t)$, and the spectrum at spectral frequency *f* (in Hz) is denoted by $\hat{w}(x,y,f)$, then according to Parseval's theorem, the following can be written

9 $$E(x,y) = \int_{ - \infty }^{ + \infty } {\left[ {w(x,y,t)} \right]^{2} {\text{d}}t} = 2\int_{0}^{ + \infty } {\left| {\hat{w}(x,y,f)} \right|^{2} } {\text{d}}f,$$

where *E* is ‘vibrational energy’ of the ground surface at (*x*, *y*). Vibration decay with distance *y* from the track central line, *DG*(*y*) in dB, can be defined as.

10 $$\begin{aligned} DG(y) & = - 10\lg \left[ {E(0,y)/E(0,0)} \right] \\ & = - 10\lg \left[ {\frac{{\int_{0}^{\infty } {\left[ {w(0,y,t)} \right]^{2} {\text{d}}t} }}{{\int_{0}^{\infty } {\left[ {w(0,0,t)} \right]^{2} {\text{d}}t} }}} \right] = - 10\lg \left[ {\frac{{\int_{0}^{\infty } {\left| {\hat{w}(0,y,f)} \right|^{2} } {\text{d}}f}}{{\int_{0}^{\infty } {\left| {\hat{w}(0,0,f)} \right|^{2} } {\text{d}}f}}} \right]. \\ \end{aligned}$$

**Figure 21:**
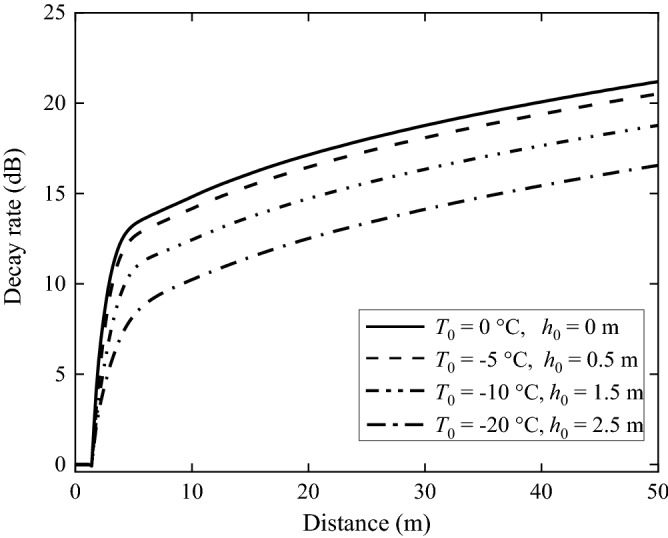
The decay rate of the ground along the direction perpendicular to the track.

Equation (10) gives the decay (compared to the level at the track centre) in overall level rather than that of each frequency component. Vibration decays of the grounds calculated according to Eq. (10) are shown in Fig. 21. It can be seen from Fig. 21 that, for all the grounds, the decay is nearly null within the track width; however, vibration is decaying with distance away from the track and the decay is much quicker near the track than further away from the track. The lower the temperature, the smaller is the decay.

(3) Only the effect of freezing temperature on fastener is considered.

**Figure 22:**
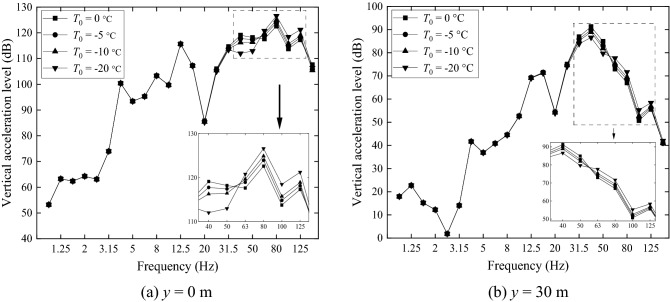
Ground vibration acceleration level in one-third octave bands at *x* = 0, (**a**) *y* = 0 m and (**b**) *y* = 30 m. Only the dependence of fastener stiffness and damping on temperature is considered.

Now consideration is only given to temperature-dependence of the fastener stiffness and damping (Table 3) by keeping the parameters of all the grounds the same as the non-frozen ground. Ground surface vibration at *x* = 0, *y* = 0 (Fig. 22a) and *x* = 0, *y* = 30 m (Fig. 22b) are plotted in Fig. 22. Figure 22a shows that, for frequencies below 31.5 Hz, changes in fastener stiffness and damping do not have a significant effect on ground vibration. This may be explained by two reasons: the first is that at low frequencies, wheel/rail roughness-induced wheel/rail forces are determined mainly by the vehicle and less affected by the track; the second is that at low frequencies, ground vibration is influenced more by the track mass than by the track stiffness. For higher frequency however, the story is different. At these frequencies, the track stiffness in the vertical direction plays a more important role in ground vibration. The freezing atmosphere temperature generates a higher fastener stiffness, which enhances the coupling between the rail and the rest of the track/ground system (slab-base-ground) and weakens the vibration isolation effect of the fastener systems, leading to higher vibration levels for the frozen grounds.

**Figure 23:**
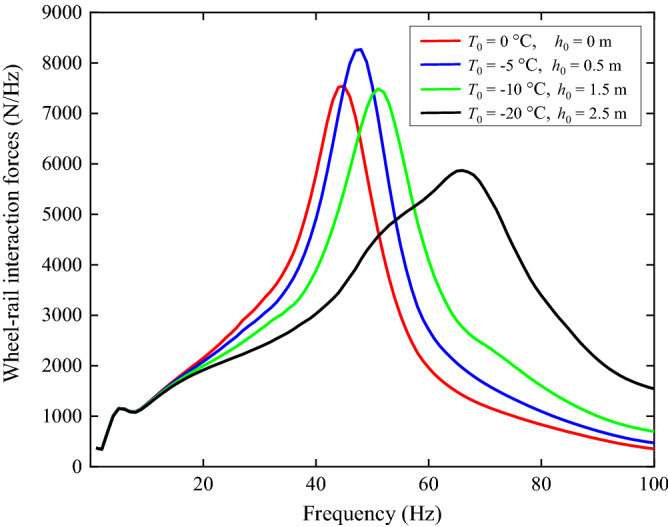
The spectra of wheel-rail interaction forces of the first wheelset.

A comparison of Fig. 18a, Fig. 19a and Fig. 22a (for ground vibration under the track) indicates that, soil-freezing has an effect across all the frequencies considered while fastener-freezing is more important at high frequencies (above 40 Hz), covering the P2-force frequency, which is the resonance frequency of the unsprung mass and the track mass vibrating on the elastic foundation of the track and can cause damage to the vehicle and track system^50,51^. The spectra of wheel-rail interaction forces of the first wheelset for different freezing temperatures are shown in Fig. 23. The lower freezing temperature (higher stiffness of the fastener system) results in a higher P2-force frequency and the P2-force frequencies are all above 40 Hz in Fig. 23, demonstrating the significant effect of fastener-freezing at high frequencies. At 40 Hz and 50 Hz in Fig. 22, the displacements of ground for *T*_0_ = − 20 °C is lower than other three cases due to the smaller wheel-rail interaction forces (black line in Fig. 23) at these frequencies while above 60 Hz, the story is quite different because the wheel-rail interaction forces for *T*_0_ = − 20 °C is largest. Comparing Fig. 19 with Fig. 22, for the observation position 30 m away from the track, it is evident that the effect of soil-freezing is much more important than fastener-freezing.

#### Z-weighted overall acceleration level

**Figure 24:**
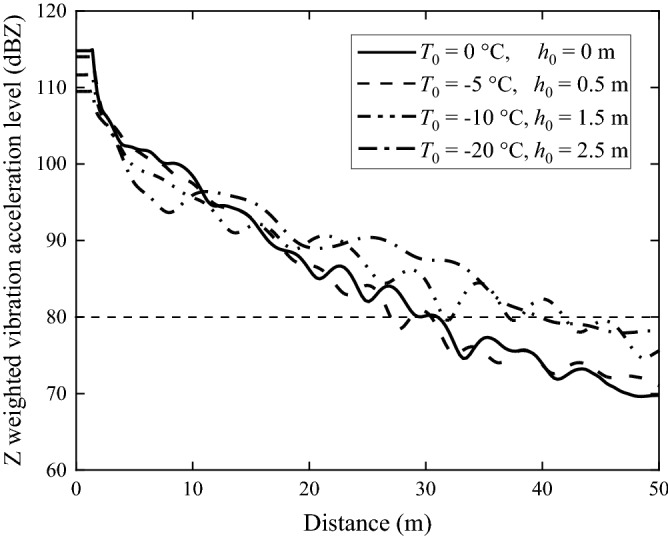
Z-weighted vibration acceleration level along the distance perpendicular to the track direction.

Z-weighted vibration levels are normally used to assess the impact of train-induced ground vibration, as described in the Chinese Standard of environmental vibration in urban area^52^. Figure 24 gives the Z-weighted overall (0–80 Hz. Weights defined in ISO 2631-1-1985 are used) vibration acceleration level as function of the distance from the track central line. In general, the vibration acceleration level decreases significantly with distance and that of the non-frozen ground decays more rapidly than the frozen ones. For the position within 10 m from the track, the response of frozen ground is well below non-frozen ground because of its higher Young’s modulus while well away from the track, the vibration level of the frozen ground is much stronger than that of the non-frozen one, mainly due to the much-reduced loss factor of the frozen ground. According to Ref.^52^, the Z-weighted vibration acceleration level should not exceed 80 dBZ beyond 30 m of the track central line. At *y* = 30 m, the Z-weighted vibration acceleration levels of the grounds with surface temperature equal to 0 °C and − 5 °C approximately reach the limit value for the analysed ground. However, when ground surface temperature drops to − 10 °C and below, the limit will be exceeded, demonstrating that the effects of soil- and fastener pad-freezing on train-induced ground vibration in frozen regions should receive more attentions. It is worth noting that the conclusions reached in this paper, are basically consistent with those in Ref.^33^, which compared the dynamic characteristics of train-induced ground vibration in summer and winter through the field experiments.

## Conclusion

In this paper, an investigation is carried out into the effects of soil and fastener-freezing on ground vibrations induced by high-speed trains. Based on the well-established relationships between soil mechanical properties and freezing temperature, a frozen ground is shown to be still represented by a layered ground and therefore, previously developed models for predicting ground vibration generated by a train running along a track resting on a layered ground can be readily applied. The layered ground is generated by dividing the soil within the frozen depth into a number of *N* sub-layers. *N* is determined based on that a further increase in the number of sub-layers does not change the dispersion curves and surface responses to harmonic load. The temperature-dependent properties of the fastener system are obtained through experiments.

Results show that, a lower freezing temperature causes higher Young’s modulus, smaller Poisson’s ratio and lower loss factor for the soil.Due to the higher Young’s modulus, a frozen ground is shown to vibrate not as strongly as the non-frozen counterpart near the track and the critical speed of track/ground also shifts to a higher one. However, since the frozen ground is much less damped, it vibrates at a higher level where sufficiently away from the track. It is also shown that, differences in ground vibration level between a frozen ground and a non-frozen ground are mainly caused by differences in soil properties. The results not only demonstrate the problem of train-induced ground vibration in frozen regions can be more serious but also remind researchers and railway engineers to consider the frozen effects of ground and fasteners and seasonal dependence during the plan for railways, evaluation of ground vibration, design of control measures.

## Data Availability

All data generated or analysed during our manuscript are included in the paper.
